# Evaluation of Equivalent Keratometry Readings Obtained by Pentacam HR (High Resolution)

**DOI:** 10.1371/journal.pone.0150121

**Published:** 2016-03-07

**Authors:** Yanjun Hua, Xiaolan Zhang, Tor Paaske Utheim, Jinhai Huang, Chao Pan, Weina Tan, Qinmei Wang

**Affiliations:** 1 Department of Ophthalmology, Shanghai Jiao Tong University Affiliated Sixth People's Hospital, Xuhui District, Shanghai, China; 2 Department of Geriatrics, Shanghai Eighth People's Hospital, Xuhui District, Shanghai, China; 3 National Centre for Optics, Vision and Eye Care, University College South East Norway, Kongsberg, Norway; 4 Department of Ophthalmology, Vestre Viken Hospital Trust, Drammen, Norway; 5 Department of Medical Biochemistry, Oslo University Hospital, Oslo, Norway; 6 School of Optometry and Ophthalmology and Eye Hospital, Wenzhou Medical University, Wenzhou, China; University of Florence, ITALY

## Abstract

**Purpose:**

To assess the repeatability of Equivalent Keratometry Readings (EKRs) obtained by the Pentacam HR (high resolution) in untreated and post-LASIK eyes, and to compare them with the keratometry (K) values obtained by other algorithms.

**Methods:**

In this prospective study, 100 untreated eyes and 71 post-LASIK eyes were included. In the untreated group, each eye received 3 consecutive scans using the Pentacam HR, and EKR values in all central corneal zone, the true net power (K_net_) and the simulated K (SimK) were obtained for each scan. In the post-LASIK group, each eye received subjective refraction and 3 consecutive scans with the Pentacam HR preoperatively. During the 3-month post-surgery exam, the same examinations and the use of an IOLMaster were conducted for each eye. The EKRs in all zone, the K_net_, the mean K (K_m_) by IOLMaster and the K values by clinical history method (K_CHM_) were obtained. The repeatability of the EKRs was assessed by the within-subject standard deviation (S_w_), 2.77S_w_, coefficient of variation (CV_w_) and intraclass correlation coefficient (ICC). The bonferroni corrected multiple comparisons were performed to analyze the differences among the EKRs and K values calculated by other algorithms within the 2 groups. The 95% limits of agreement (LoA) were calculated.

**Results:**

The EKR values in all central corneal zone were repeatable in both the untreated group (S_w_≦0.19 D, 2.77S_w_≦0.52 D, CV_w_≦1%, ICC≧0.978) and the post-LASIK group (S_w_≦0.22 D, 2.77S_w_≦0.62 D, CV_w_≦1%, ICC≧0.980). In the untreated group, the EKR in 4mm zone was close to SimK (*P* = 1.000), and the 95% LoA was (-0.13 to 0.15 D). The difference between K_net_ and SimK was -1.30±0.13 D (95% LoA -1.55 to -1.55 D, *P*<0.001). In the post-LASIK group, all the EKRs were significantly higher than K_CHM_ (all *P*<0.001). The differences between the EKR in 4mm zone and K_CHM_, the EKR in 7mm zone and K_CHM_, K_net_ and K_CHM_, K_m_ and K_CHM_, SimK and K_net_ were 0.64±0.50 D (95% LoA, -0.33 to 1.62 D), 1.77±0.88 D (95% LoA, 0.04 to 3.51 D), -0.98±0.48 D (95% LoA, -1.92 to -0.04 D), 0.64±0.53 D (95% LoA, -0.40 to 1.68 D), and 1.73±0.20 D (95% LoA, 1.33 to 2.13 D), respectively.

**Conclusions:**

The EKRs obtained by the Pentacam HR were repeatable in both untreated eyes and post-LASIK eyes. Compared to the total corneal power obtained by the clinical history method, the EKR values generally overestimated the total corneal power in post-LASIK eyes. So, further calibrations for the EKR values should be conducted, before they were used for the total corneal power assessment in post-LASIK eyes.

## Introduction

The accurate assessment of the total corneal power in eyes after corneal refractive surgery is essential for the prediction of intraocular lens (IOL) power[1–3]. Currently, there are several methods to assess total corneal power after corneal refractive surgery, which can be divided into two categories. One requires preoperative data, including the clinical history method (K_CHM_)[4] and double-K method[5, 6]. The other category does not require preoperative data and includes the rigid contact lens method[7], the Maloney method[8–10], Shammas formula[11, 12] and the BESSt formula[13].

The Holladay’s Equivalent Keratometry Readings (EKRs) obtained by the Pentacam HR belongs to the latter[14]. However, the precision of EKRs for the assessment of total corneal power after corneal refractive surgery is still controversial. The repeatability of the EKR (4.5 mm central corneal zone) from untreated eyes has been shown to be suboptimal[15]. Additionally, it was reported that the EKRs were not accurate for the prediction of IOL power in both untreated eyes and postoperative eyes[16].

The purpose of the present study was to assess the repeatability of the EKR in all central corneal zones obtained by the Pentacam HR. In untreated eyes, we compared the EKRs in all zones with the simulated keratometry (SimK) obtained by the Pentacam HR, and in post-LASIK eyes, we mainly compared the EKRs in all zones with K_CHM_.

## Subjects and Methods

The present study was conducted at the Eye Hospital of Wenzhou Medical University (Wenzhou, China) between August and December 2011. The study was performed in accordance with the principles stated in the Declaration of Helsinki and was approved by the Office of Research Ethical Committee, Eye Hospital of Wenzhou Medical University. All subjects were informed of the purpose of the research and provided signed informed consent. Two groups were included in this study, the untreated group and the post-LASIK group. For the untreated group, inclusion criteria were healthy subjects aged 18 to 40 years old, without communication or cooperation disorders. All the subjects had the following qualities: 1) a preoperative best corrected visual acuity of 1.0 or more, 2) a corneal astigmatism of less than 2.0 diopter (D), and 3) an intraocular pressure range of 10 mmHg to 21 mmHg. The exclusion criteria included the following: 1) history of ocular pathology, 2) history of corneal or intraocular trauma, 3) previous ocular surgery, 4) the wearing of hard contact lenses within 4 weeks or soft contact lenses within 2 weeks, and 5) severe dry eye (tear film break-up time shorter than 5 seconds). For the post-LASIK group, besides the inclusion and exclusion criteria stated above, the postoperative uncorrected visual acuity was no less than 1.0 without corneal opacities.

In the untreated group, subjects received 3 consecutive scans using the Pentacam HR (Oculus, Germany) after a routine ophthalmic examination (including subjective refraction, slit lamp examination, fundus examination and intraocular pressure). In addition to the preoperative exams of the untreated group, the post-LASIK group received exams more than three months after the surgery, including the following: subjective refraction, the Pentacam HR (with 3 consecutive scans) and IOLMaster (Carl Zeiss). The Pentacam HR was performed in a dark room. Subjects were told to place their chin on the chinrest, focus on the indicator, and widely open their eyes after blinking. The Pentacam HR would take pictures automatically within 2 seconds. When the quality specification screen displayed “OK”, the result was considered valid. Three valid scans for each eye were obtained. The mean keratometry (K_m_) from post-LASIK eyes was obtained by the IOLMaster. In the post-LASIK group, the surgery was performed using the Allegretto laser system (Lumenis, Inc., USA). The diameters of optical zone ranged from 6.0 mm to 7.0 mm during the surgery.

### Parameters for assessment of corneal power in the Pentacam HR

The anterior and posterior central corneal curvature, within a certain diameter (in meter), were defined as r_anterior_ and r_posterior_, respectively. The refractive index of air (n_0_) is 1.000. The standard corneal refractive index (n) is 1.3375. The real corneal refractive index (n_1_) is 1.376. The aqueous refractive index (n_2_) is 1.336. The ratio of posterior and anterior curvature from an untreated cornea (R_1_) is 0.822[17–19]. The ratio of simulated keratometry and anterior corneal power (R_2_) is (1.3375–1.000)/(1.376–1.000) = 0.8976.

The SimK was calculated based on the anterior central corneal curvature within certain range and the standard corneal refractive index as follows:

$$\text{SimK}=(\text{n}-{\text{n}}_{0})/{\text{r}}_{\text{anterior}}.$$

The true net power (K_net_)[20] represents the sum of the anterior and posterior corneal power and is calculated using the following formula:

$${\text{K}}_{\text{net}}=({\text{n}}_{1}-{\text{n}}_{0})/{\text{r}}_{\text{anterior}}+({\text{n}}_{2}-{\text{n}}_{1})/{\text{r}}_{\text{posterior}}.$$

The EKR[14] was advanced by Holladay and his colleagues specifically for the evaluation of total corneal power after corneal refractive surgery and has been obtained in Pentacam HR since 2006. In the Pentacam HR’s software interface “Holladay EKR Detail Report” values are given within 1 mm, 2 mm, 3 mm, 4 mm, 4.5 mm, 5 mm, 6 mm and 7 mm of the central corneal diameter, respectively. The values were calculated using the following formula:

$$\text{EKR}\;(\text{D})=({\text{n}}_{1}-1)/{\text{r}}_{\text{anterior}}+(\text{n}-1)(1-1/{\text{R}}_{2})\times {\text{R}}_{1}/{\text{r}}_{\text{posterior}}$$

This formula above, however, can be simplified to:

$$\text{EKR}\;(\text{D})\;=\;0.376/{\text{r}}_{\text{anterior}}-0.03165/{\text{r}}_{\text{posterior}}.$$

In this study, the mean EKR within 1 mm, 2 mm, 3 mm, 4 mm, 4.5 mm, 5 mm, 6 mm and 7 mm zones were calculated and were abbreviated as EKR1, EKR2, EKR3, EKR4, EKR4.5, EKR5, EKR6 and EKR7, respectively.

The following three parameters are needed for the calculation of K_CHM_[4]: 1) the preoperative spherical equivalent (SEQ_pre_) at corneal plane, 2) the postoperative spherical equivalent (SEQ_post_) at the corneal plane and 3) the preoperative simulated keratometry (preK). First, the amount of refraction change in diopters before and after the surgery is calculated using this formula:

$$\Delta \;\text{SEQ}\;=\;{\text{SEQ}}_{\text{pre}}\;-\;{\text{SEQ}}_{\text{post}}.$$

Second, the K_CHM_ is calculated based on the following formula:

$${\text{K}}_{\text{CHM}}\;=\;\text{preK}\;-\;\Delta \;\text{SEQ}.$$

In this study, preK was obtained by the Pentacam HR, and we regarded the K_CHM_ as the benchmark to assess the EKR, SimK and K_net_ obtained by the Pentacam HR in the post-LASIK group.

### Statistical analysis

All data were entered into an Excel spreadsheet (Microsoft Excel 2010 Crop.), and MedCalc Software (Vision11.4.2.0, MedCalc, Inc.) was used for statistical analysis. The results were expressed as the mean ± standard deviation (SD). A *P* value less than 0.05 was considered statistically significant. The normality of all data distributions was confirmed by the Kolmogorov-Smirnov test (all *P*>0.05), and parametric statistical tests were used for data analysis. Repeated measures ANOVA was applied to analyze the repeatability of EKR in different zones and the SimK and K_net_ in both the untreated and post-LASIK groups_._ The within-subject SD (S_w_) of three consecutive measurements were calculated. The repeatability limit was defined as 2.77S_w_, which means an interval within which 95% of the differences between measurements are expected to lie. The within-subject coefficient of variance (CV_w_) was calculated as the ratio of the S_w_ to the overall mean. Using the CV_w_, data with different means can be compared with each other. A lower CV_w_ is associated with higher repeatability. The intraclass correlation coefficients (ICCs) were based on the analysis of variance for a two-way mixed-effects model with an absolute agreement for consistency of individual measurements and the 95% confidence interval (CI) was calculated. The ICC (ranging from 0 to 1) assesses the consistency for data sets of repeated measurements. As the ICC nears a value of 1, the measurement consistency increases, and a value more than 0.9 indicates acceptable clinical reliability[21]. The repeated measures analysis of variance with the Bonferroni corrected multiple comparisons was applied to compare the EKRs, SimK and K_net_ in the untreated group, and the EKRs, SimK, K_net_ and K_CHM_ in post-LASIK group. The 95% limits of agreement (LoA) were calculated.

## Results

In the untreated group, 100 eyes (47 men and 53 women) were included, and the mean manifest spherical equivalent refraction was -3.23±1.76 D. In the post-LASIK group, 71 eyes (24 men and 19 women) were included. The mean manifest spherical equivalent refraction before the surgery and at least three months after surgery were -5.01±1.91 D (range -9.50 D to -1.50 D) and 0.03±0.38 D (range -1.50 D to 0.88 D), respectively. The mean amount of refraction change in diopters before and after the surgery was 5.05±1.81 D (range 1.25 to 8.88 D).

### The repeatability of the EKRs in different zones, SimK and K_net_ (Table 1)

In both the untreated group and the post-LASIK group, all the S_w_, 2.77S_w_ and CV_w_ values were less than 0.22 D, 0.62 D and 1%, respectively, and all ICCs were higher than 0.978. The Pentacam HR performed high repeatability of all the EKRs, SimK and K_net_ values from both untreated eyes and post-LASIK eyes.

**Table 1:** The repeatability of the EKRs, SimK and K_net_ in untreated eyes (n = 100) and post-LASIK eyes (n = 71).

Parameter	Mean(D)±SD	S_w_(D)	2.77S_w_(D)	CV_w_(%)	ICC(95%CI)
Untreated eyes					
EKR1	43.40±1.28	0.19	0.52	0.43	0.978(0.970 to 0.985)
EKR2	43.39±1.28	0.16	0.44	0.36	0.985(0.979 to 0.989)
EKR3	43.42±1.28	0.11	0.31	0.26	0.992(0.989 to 0.994)
EKR4	43.50±1.29	0.08	0.22	0.18	0.996(0.995 to 0.997)
EKR4.5	43.56±1.29	0.07	0.20	0.17	0.997(0.995 to 0.998)
EKR5	43.65±1.30	0.06	0.17	0.14	0.998(0.997 to 0.999)
EKR6	43.87±1.33	0.05	0.13	0.11	0.999(0.998 to 0.999)
EKR7	44.14±1.36	0.05	0.13	0.10	0.999(0.998 to 0.999)
SimK	43.48±1.28	0.08	0.23	0.19	0.996(0.994 to 0.997)
K_net_	42.18±1.26	0.10	0.26	0.23	0.994(0.992 to 0.996)
Post-LASIK eyes					
EKR1	39.48±1.59	0.22	0.62	0.57	0.980(0.971 to 0.987)
EKR2	39.31±1.61	0.16	0.45	0.41	0.990(0.985 to 0.993)
EKR3	39.13±1.65	0.12	0.33	0.30	0.995(0.993 to 0.997)
EKR4	39.04±1.67	0.09	0.24	0.22	0.997(0.996 to 0.998)
EKR4.5	39.06±1.67	0.08	0.23	0.21	0.998(0.997 to 0.998)
EKR5	39.14±1.66	0.07	0.20	0.19	0.998(0.997 to 0.999)
EKR6	39.53±1.63	0.07	0.20	0.18	0.998(0.997 to 0.999)
EKR7	40.17±1.60	0.07	0.18	0.16	0.998(0.998 to 0.999)
SimK	39.15±1.69	0.09	0.24	0.22	0.997(0.996 to 0.999)
K_net_	37.42±1.73	0.09	0.24	0.23	0.997(0.996 to 0.998)

EKR1/2/3/4/4.5/5/6/7, Equivalent Keratometry Readings in 1 mm/2 mm/3 mm/4 mm/4.5 mm/5 mm/6 mm/7 mm zone; SimK, mean Simulated Keratometry obtained by Pentacam HR; K_net_, the true net power in 3mm central corneal zone obtained by Pentacam HR; CV_w_, within-subject coefficient of variation; S_w_: within-subject standard deviation; ICC, intraclass correlation coefficient; 95% CI, 95% confidence interval.

### Comparison of the EKRs, SimK, K_net_ and K_CHM_ (Table 2)

**Fig 1:**
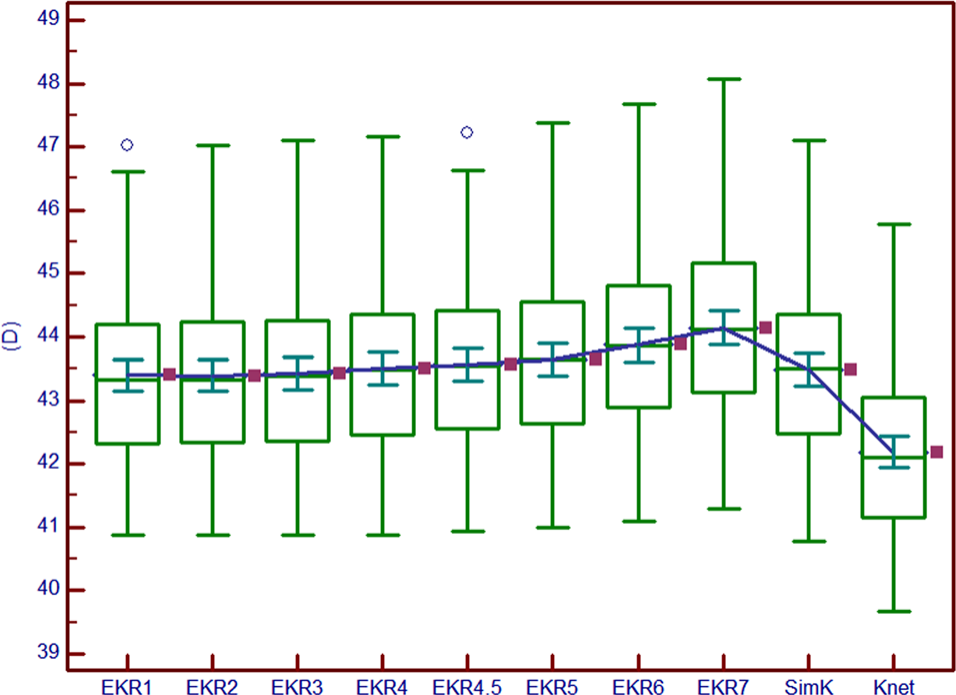
Multiple variables graph in the untreated group. EKR1/2/3/4/4.5/5/6/7, Equivalent Keratometry Readings in 1 mm /2 mm/3 mm/4 mm/4.5 mm/5 mm/6 mm/7 mm zone; SimK, mean Simulated Keratometry obtained by Pentacam HR; K_net_, the true net power obtained by Pentacam HR.

In the untreated group, among all the EKRs, EKR2 (43.39±1.28 D) was the smallest, but not significantly smaller than EKR1 (43.40±1.28 D, P = 1.000). EKR2 was, however, significantly lower than all the other EKR (P<0.05) and SimK (P<0.001), and significantly higher than K_net_ (P<0.001). SimK was 43.48±1.28 D. Comparing all the EKR to SimK, EKR1, EKR2 and EKR3 were significantly lower than SimK. EKR4.5, EKR5, EKR6 and EKR7 were significantly higher than SimK. EKR4 was close to SimK (*P* = 1.000), and the 95% LoA was -0.13 to 0.15 D (Fig 1). The difference between K_net_ and SimK was -1.30±0.13 D (95% LoA, -1.55 to -1.04 D).

**Table 2:** The differences of the EKRs, SimK, K_net_ and K_CHM_ in untreated and Post-LASIK eyes.

Parameter Pairing	Mean Difference (D) ± SD	95%CI (D)	95% LoA (D)	P^a^
Untreated eyes				
EKR1-SimK	-0.09±0.20	-0.15 to -0.02	-0.47 to 0.30	<0.001
EKR2-SimK	-0.09±0.15	-0.14 to -0.04	-0.38 to 0.20	<0.001
EKR3-SimK	-0.06±0.10	-0.10 to -0.03	-0.26 to 0.13	<0.001
EKR4-SimK	0.01±0.07	0.00 to 0.03	-0.13 to 0.15	1.000
EKR4.5-SimK	0.08±0.07	0.06 to 0.10	-0.05 to 0.21	<0.001
EKR5-SimK	0.17±008	0.14 to 0.19	0.02 to 0.31	<0.001
EKR6-SimK	0.39±0.12	0.35 to 0.43	0.16 to 0.62	<0.001
EKR7-SimK	0.66±0.17	0.60 to 0.72	0.33 to 0.99	<0.001
K_net_ -SimK	-1.30±0.13	-1.34 to -1.25	-1.55 to -1.04	<0.001
Post-LASIK eyes				
EKR1-K_CHM_	1.08±0.76	0.77 to 1.40	-0.40 to 2.56	<0.001
EKR2-K_CHM_	0.93±0.66	0.64 to 1.19	-0.37 to 2.20	<0.001
EKR3-K_CHM_	0.73±0.54	0.51 to 0.96	-0.33 to 1.80	<0.001
EKR4-K_CHM_	0.64±0.50	0.44 to 0.85	-0.33 to 1.62	<0.001
EKR4.5-K_CHM_	0.67±0.51	0.46 to 0.88	-0.33 to 1.66	<0.001
EKR5-K_CHM_	0.75±0.54	0.52 to 0.98	-0.31 to 1.82	<0.001
EKR6-K_CHM_	1.13±0.68	0.85 to 1.42	-0.21 to 2.47	<0.001
EKR7-K_CHM_	1.77±0.88	1.40 to 2.14	0.04 to 3.51	<0.001
SimK-K_CHM_	0.75±0.51	0.54 to 0.96	-0.26 to 1.75	<0.001
K_net_-K_CHM_	-0.98±0.48	-1.18 to -0.78	-1.92 to -0.04	<0.001
K_m_-K_CHM_	0.64±0.53	0.42 to 0.86	-0.40 to 1.68	<0.001
SimK-K_net_	1.73±0.20	1.65 to 1.81	1.33 to 2.13	<0.001
SimK-K_m_	0.11±0.32	-0.02 to 0.24	-0.51 to 0.73	0.324

EKR1/2/3/4/4.5/5/6/7, Equivalent Keratometry Readings in 1 mm/2 mm/3 mm/4 mm/4.5 mm/5 mm/6 mm/7 mm zone; SimK, mean Simulated Keratometry obtained by Pentacam HR; K_net_, the true net power obtained by Pentacam HR; Km, mean keratometry obtained by IOLMaster; K_CHM_, total corneal power obtained by clinical history method; 95% CI, 95% confidence interval; 95% LoA, 95% limits of agreement

^a^, Bonferroni corrected multiple comparisons.

**Fig 2:**
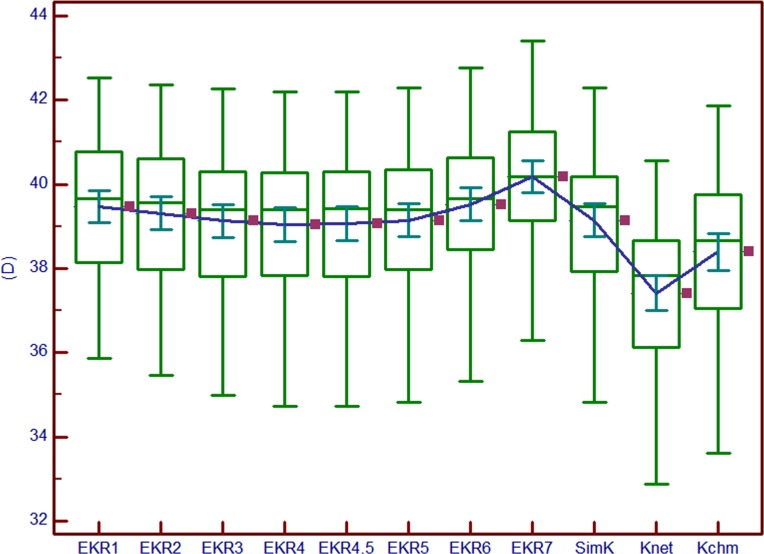
Multiple variables graph in the post-LASIK group. EKR1/2/3/4/4.5/5/6/7, Equivalent Keratometry Readings in 1 mm/2 mm/3 mm/4 mm/4.5 mm/5 mm/6 mm/7 mm zone; SimK, mean Simulated Keratometry obtained by Pentacam HR; K_net_, the true net power obtained by Pentacam HR; K_CHM_, total corneal power obtained by clinical history method.

In the post-LASIK group, it indicated that the trend from EKR1 to EKR7 gradually decreased to EKR4 (39.04±1.67 D) as the minimum, then increased to EKR7 (Fig 2). There was no statistical significance between EKR4 and EKR4.5 (*P* = 0.396), EKR3 and EKR4.5 (*P* = 0.559). K_CHM_ was 38.40±1.86 D. The differences between EKR4 and K_CHM_, EKR7 and K_CHM_, K_net_ and K_CHM_, K_m_ and K_CHM_, SimK and K_net_ were 0.64±0.50 D (95% LoA, -0.33 to 1.62 D), 1.77±0.88 D (95% LoA, 0.04 to 3.51 D), -0.98±0.48 D (95% LoA, -1.92 to -0.04 D), 0.64±0.53 D (95% LoA, -0.40 to 1.68 D), and 1.73±0.20 D (95% LoA, 1.33 to 2.13 D), respectively. SimK was equal to K_m_ (95% LoA -0.51 to 0.73 D, *P* = 0.324, Fig 2).

## Discussion

The EKR assessment, advanced by Holladay and co-workers, was used to assess the total corneal power after corneal refractive surgery. In the study by Holladay et al[14], the Pentacam was used to obtain the anterior and posterior corneal power of 100 post-LASIK eyes and the formula (EKR(D) = 0.376/r_anterior_ − 0.03165/r_posterior_) was used to calculate EKR from different central corneal zones (from 0.5 mm to 8 mm diameter, each 0.5 mm to an order). Holladay et al observed that the EKR of the 4.5 mm diameter zone was close to the total corneal power obtained by the clinical history method. Comparing the EKR4.5 to the back calculated total corneal power in 41 eyes that received both RK and cataract surgery, small errors were found. Based on these results, Holladay et al recommended EKR4.5 to be used for the assessment of corneal power after corneal refractive surgery.

Thus far, there have been very few studies concerning the repeatability of the EKRs. McAlinden et al[15] used the Pentacam HR to measure 100 untreated eyes and found that the repeatability of EKR maps and the Holladay report Equivalent K1, K2 and Km (EKR4.5) were poor. In contrast, in the present study, EKRs in all zones from untreated eyes were repeatable. According to McAlinden et al, only the EKR in 4.5 mm was evaluated, and we evaluated the EKRs in all central corneal zones. Moreover, this study was the first to report the repeatability of EKRs in all zones in post-LASIK eyes, and the results for the EKRs were repeated in all central corneal zones.

Interestingly, in the post-LASIK group, it indicated that the trend from EKR1 to EKR7 gradually decreased to EKR4 as the minimum, then increased to EKR7 (Fig 2). This result was different from the studies by Falavarlani et al[22] (35 eyes after PRK) and Savini et al[23] (16 eyes after myopic laser correction) in which the values gradually increased from EKR1 to EKR4.5. According to Falavarlani et al, the EKR values increased from 40.43±2.04 D to 40.91±1.82 D, and according to Savini et al, they increased from 37.46±1.77 D to 38.40±1.13 D. The difference might be explained by the use of the different surgical approaches and laser systems used during surgery. In our study, only post-LASIK eyes were included, while Falavarlani et al only included post-PRK eyes. Savini et al included eyes after LASIK, PRK and LASEK.

Currently, there is no generally accepted device that can exactly measure and calculate the total corneal power after corneal refractive surgery. The clinical history method, which was presented in 1989 by Holladay et al, has been regarded as the gold standard in several published studies[13, 14, 22–24]. In our study, K_CHM_ was smaller than all the EKRs, and compared to EKR4 (the smallest among all the EKRs), the difference was 0.64±0.50 D (*P*<0.001). The K_m_ obtained by the IOLMaster was equal to EKR4 and similar to SimK. We compared our study with Falavarjani et al[22] and Savini et al[23]. The K_CHM_, SimK and EKR in our study were higher than in Savini et al, but lower than in Falavarjani et al. One possibility may be due to the different preoperative refraction of the subjects. In our study, the mean preoperative spherical equivalent was -5.01±1.91 D (range -9.50 D to -1.50 D). According to Savini et al and Falavarjani et al, the values were -5.10±1.40 D, and -3.46±1.16 D (range -6.00 D to -1.50 D), respectively. In the event of similar mean corneal power, a higher refractive error will lead to a larger amount of surgical correction. This leads to a flatter cornea with smaller postoperative corneal power. Based on all three studies, we speculate that the EKR overestimates the corneal power in post-LASIK eyes. Two observations support this notion. First, in our study the EKR4 (the smallest among all the EKRs) was 0.6 to 0.7 D higher than the gold standard K_CHM_. Second, the SimK, K_m_, EKR4 and EKR4.5 in our study were very close to each other (the same results were shown by Savini et al and Falavajani et al). As SimK and K_m_ were calculated by the same formula (SimK = 1.3375/r_anterior_), which explains why no statistical significance was observed between SimK and K_m_. For untreated eyes, we could obtain accurate results using this formula; however, for eyes after corneal refractive surgery, because of the change of ratio between the posterior and anterior corneal curvature, the corneal power was overestimated[25, 26]. And in our study, EKR4 (the minimum of EKRs) was equal to K_m_, and similar to SimK. Therefore, we conclude that EKR4 also overestimates the corneal power in post-LASIK eyes.

In our study, SimK was 1.30±0.13 D higher than K_net_ in the untreated group, and in the post-LASIK group the difference increased to 1.68±0.42 D. The difference was attributed to two factors. First, the two parameters were calculated using a different corneal refractive index based on different assumptions. For SimK, the standard corneal refractive index (1.3375) was adopted considering the cornea as a thin lens; for K_net_, the real corneal refractive index (1.376) and aqueous refractive index (1.336) were adopted. Savini et al[27] used the Pentacam to measure 71 preoperative eyes, and found that SimK was 1.25 D higher than K_net_, which was very close to our study (1.30±0.13 D). Thus, we believe that this factor might cause the deviation of 1.2 to 1.3 D. It should be noted that K_net_, which is the sum of anterior and posterior corneal power (without consideration of CCT), was different from the total corneal power based on the Gaussian thick lens formula (with consideration of CCT). The difference, which was between 0.12 to 0.25 D[20], could be regarded as acceptable in the clinic. Second, for post-LASIK eyes the ratio of the posterior and anterior corneal curvature is considerably different from of the Gullstrand model eye value of 0.883. Therefore, the standard corneal refractive index based on the Gullstrand model eye caused the wrong calculation, explaining the deviation of approximately 0.4 D.

There were two limitations to our study. First, we only studied untreated eyes and myopic post-LASIK eyes, the eyes after RK and hyperopia corneal refractive surgery were not involved. Second, the repeatability of the clinical history method was not confirmed. The clinical history method has been suggested not to be the most accurate for the prediction of IOL power after corneal refractive surgery[28].

In conclusion, the EKR obtained by the Pentacam HR were repeatable in both untreated eyes and post-LASIK eyes. Compared to the clinical history method, EKRs generally overestimated the corneal power in post-LASIK eyes. EKR4 and EKR4.5 were closest to the clinical history method, but were still overestimated by 0.6 to 0.7 D.

## Supporting Information

S1 Checklist PLOS ONE clinical studies checklist. (DOCX)

S1 Data Original data from untreated group. (XLSX)

S2 Data Original data from post-LASIK group. (XLSX)
